# The Impact of Uterine Peristalsis on Clinical Pregnancy Rates in In Vitro Fertilisation (IVF): A Systematic Review and Meta-Analysis

**DOI:** 10.7759/cureus.114760

**Published:** 2026-08-18

**Authors:** Acha Bachir Assmanni Adam, Amani Seifeldin Mahmoud Khalifa, Asma Fawzi Mohamed Al Rujaibi, Reem Badawi Hamad Yousif, Hiba Abdelraouf Hyder Mohammed, Rania Mahmoud Hamid Osman, Sundos Mahgoub Ahmed Mahmoud

**Affiliations:** 1 Obstetrics and Gynecology, Madina National Hospital, Madina, SAU; 2 Obstetrics and Gynecology, Ahfad University for Women, Khartoum, SDN; 3 Obstetrics and Gynaecology, Royal Hospital, Muscat, OMN; 4 Gynecology Oncology, Sheikh Khalifa Specialty Hospital Ras Al Khaimah, Ras Al Khaimah, ARE; 5 Preventive Medicine, Al Mairid Primary Healthcare Center, Ras Al Khaimah, ARE; 6 Obstetrics and Gynecology, Al Azhar Hospital, Riyadh, SAU; 7 Obstetrics and Gynecology, Alkharj Military Hospital, Ministry of Defense Health Services, Alkharj, SAU

**Keywords:** clinical pregnancy, embryo transfer, endometrial receptivity, in vitro fertilisation, meta-analysis, uterine contractility, uterine peristalsis

## Abstract

Uterine peristalsis - rhythmic, wave-like contractile activity of the subendometrial myometrium - has been proposed as a factor that may influence embryo implantation. Whether peristaltic frequency measured around the time of embryo transfer is associated with clinical pregnancy in in vitro fertilisation (IVF) remains incompletely resolved, and existing syntheses have pooled studies in which the exposure was not measured during the transfer cycle itself.

We conducted a systematic review and meta-analysis in accordance with the Preferred Reporting Items for Systematic Reviews and Meta-Analyses (PRISMA) 2020 statement. Eligible studies were original, peer-reviewed observational studies of women undergoing IVF or intracytoplasmic sperm injection with embryo transfer, in which uterine peristaltic frequency was quantified by ultrasound during the embryo transfer cycle and clinical pregnancy was reported by peristalsis stratum. Reviews, conference abstracts, preprints, and studies of pharmacological interventions altering contractility were excluded. Risk of bias was appraised with the Risk Of Bias In Non-randomised Studies - of Exposures (ROBINS-E) tool, and certainty of evidence with the Grading of Recommendations Assessment, Development and Evaluation (GRADE) framework. Odds ratios (ORs) were pooled using DerSimonian-Laird random-effects models. Given the small anticipated evidence base, subgroup, funnel-plot, and sensitivity analyses were prespecified as exploratory.

Four studies comprising 863 embryo transfer cycles and 371 clinical pregnancies met eligibility criteria. Clinical pregnancy occurred in 160 of 478 cycles (33.5%) with high uterine peristalsis and 211 of 385 cycles (54.8%) with low peristalsis. High peristaltic frequency was associated with approximately half the odds of clinical pregnancy (pooled OR 0.45, 95% confidence interval [CI] 0.32-0.62; P < 0.001), corresponding to 197 fewer clinical pregnancies per 1,000 cycles (95% CI 268 fewer to 120 fewer). Heterogeneity was low (I² = 19.8%; τ² = 0.023; Q = 3.74, df = 3, P = 0.29), although the 95% prediction interval (0.17-1.17) crossed unity. The association was robust to leave-one-out analysis (OR range 0.38-0.50) and to restriction to studies using a harmonised 2 waves/min threshold (OR 0.50, 95% CI 0.36-0.69; I² = 1.4%). Subgroup analyses by cycle type and exposure threshold showed no significant effect modification. Funnel plot asymmetry was not detected (Egger P = 0.27), though power was minimal. Certainty of evidence was very low.

Elevated uterine peristaltic frequency measured during the embryo transfer cycle is consistently associated with substantially reduced odds of clinical pregnancy in IVF. However, the evidence derives from four small, unblinded observational studies using non-standardised, largely post hoc frequency thresholds assessed at differing times relative to transfer, and a prediction interval crossing unity indicates that a future study could plausibly find no association. Uterine peristalsis should be regarded as a promising but unvalidated prognostic marker; clinicians cannot currently alter management on the basis of peristalsis alone, and standardised measurement protocols and prospective evaluation against live birth are prerequisites for any clinical role.

## Introduction and background

The non-pregnant human uterus is not quiescent. Ultrasonography of the subendometrial myometrium reveals rhythmic, wave-like contractile activity - uterine peristalsis - whose frequency, amplitude, and direction vary systematically across the menstrual cycle [1]. Peristaltic frequency rises through the follicular phase to a periovulatory maximum, when predominantly cervicofundal waves are thought to assist sperm transport, and then declines markedly during the luteal phase, producing a relatively quiescent uterus at the time the blastocyst would physiologically arrive [1,2]. This luteal decline is teleologically attractive: a mechanically calm cavity plausibly favours the sustained apposition between embryo and endometrium that implantation requires. Controlled ovarian hyperstimulation, however, disrupts this pattern. In a prospective within-woman comparison, peristaltic frequency was substantially higher in stimulated cycles than in the same women's natural cycles, and endometrial activity did not return to natural-cycle levels before embryo transfer despite supraphysiological progesterone concentrations [3]. Assisted reproduction may therefore deposit embryos into a uterus that is mechanically more active than the one nature intended.

This observation motivated a series of clinical studies asking whether peristaltic activity at the time of transfer is associated with outcome. In the foundational investigation, Fanchin et al. studied 220 stimulated cycles and observed a stepwise fall in clinical pregnancy across ascending frequency strata, from 53% at ≤3.0 contractions/min to 14% above 5.0 contractions/min, with frequency correlating inversely with plasma progesterone [4]. Zhu et al. extended this to 292 fresh and frozen-thawed cycles, reporting an independent association in multivariable analysis (adjusted OR 0.49, 95% CI 0.34-0.70) [5]; Chung et al. related contraction frequency before and after transfer to reduced live birth in 283 women [6]; and Javedani Masroor et al. observed a comparable gradient across 68 frozen-thawed cycles [7]. Across a quarter-century (1998-2023), four countries, and both fresh and frozen protocols, the direction of association has been consistent - although, as detailed below, the exposure was defined and timed differently in every study.

Consistency of direction, however, is not the same as sufficiency of evidence. Two prior syntheses have addressed this literature. Kuijsters et al. provided a broad narrative review with limited quantitative pooling, concluding that initially promising findings had not translated into clinical practice [8]. More recently, Vidal et al. pooled five studies and reported that women with more than two contractions per minute had roughly half the clinical pregnancy rate of those with fewer (OR 0.52, 95% CI 0.38-0.69), but with moderate heterogeneity (I² = 55%) [9]. That analysis has two features that invite re-examination. First, it applied a uniform >2 versus ≤2 contractions/min dichotomy across all pooled studies, although the largest early study reported its lowest stratum as ≤3.0 contractions/min and therefore cannot be dichotomised at 2.0 from published data [4]. Second, it included a study in which peristalsis was assessed during a natural cycle preceding in vitro fertilisation (IVF), before a mock rather than an actual transfer - a study whose authors explicitly reported no difference in peristaltic frequency between women who subsequently conceived and those who did not. The present review was undertaken to re-examine this question under a deliberately stricter exposure definition, restricting inclusion to studies measuring peristalsis during the embryo transfer cycle itself, transparently handling threshold heterogeneity through prespecified subgroup analysis, and formally appraising both risk of bias and certainty of evidence. We frame the question throughout as one of association rather than causation, since the available evidence is entirely observational. Our objective was to quantify the association between uterine peristaltic frequency measured around embryo transfer and clinical pregnancy in IVF, and to establish how much confidence the available evidence can bear.

## Review

Materials and methods

Study Design

This systematic review and meta-analysis was designed and reported in accordance with the Preferred Reporting Items for Systematic Reviews and Meta-Analyses (PRISMA) 2020 statement [10]. The review addressed a single prespecified question framed using the Population, Intervention/Exposure, Comparator, Outcome, and Study design (PICOS) structure. The review protocol was not prospectively registered in PROSPERO or another registry; this absence of prospective registration is stated explicitly here, in the interest of full PRISMA 2020 transparency, and is acknowledged again among the limitations.

Eligibility Criteria

Eligibility was defined a priori using the PICOS framework and is summarised in Table 1. In brief, we included original, peer-reviewed studies of women undergoing IVF or intracytoplasmic sperm injection culminating in embryo transfer, in which uterine peristaltic or contraction frequency was quantified by ultrasound during the embryo transfer cycle and in which clinical pregnancy was reported separately for higher- and lower-frequency strata, permitting construction of a two-by-two table.

**Table 1:** Eligibility criteria structured according to the PICOS framework IVF: in vitro fertilisation; ICSI: intracytoplasmic sperm injection; IUI: intrauterine insemination; PICOS: Population, Intervention/Exposure, Comparator, Outcome, and Study design

Domain	Inclusion criteria	Exclusion criteria
Population	Women undergoing IVF or ICSI with embryo transfer	Non-IVF conception; IUI or natural-cycle-only cohorts; donor-oocyte-only cohorts
Exposure	Uterine peristaltic frequency quantified by ultrasound during the embryo transfer cycle	Peristalsis measured in a preceding or non-transfer cycle; mock transfer only; amplitude or direction without frequency
Comparator	Lower peristaltic frequency stratum, at the study's reported threshold	No internal comparator stratum
Outcome	Clinical pregnancy (ultrasound-confirmed gestational sac) reported by peristalsis stratum	Biochemical pregnancy only; outcomes not reported by stratum
Study design	Original peer-reviewed prospective or retrospective cohort studies	Reviews, editorials, case reports, conference abstracts, preprints, animal studies, interventional studies of contractility-modifying agents

Three exclusions warrant emphasis. First, narrative reviews, systematic reviews, editorials, commentaries, conference abstracts without full publication, and preprints lacking peer review were excluded, since these do not constitute primary evidence. Second, studies evaluating pharmacological or mechanical interventions intended to modify contractility - including oxytocin receptor antagonists, progestogens administered specifically to alter peristalsis, prostaglandin synthetase inhibitors, and nitric oxide donors - were excluded, because the exposure in such studies is the intervention rather than spontaneous peristalsis. Third, and distinguishing this review from earlier syntheses, studies in which peristalsis was measured outside the embryo transfer cycle - for example during a preceding natural cycle or in association with a mock transfer - were excluded from the primary analysis, on the grounds that peristaltic activity is known to differ substantially between natural and stimulated cycles [3] and that exposure measured in a different cycle is not the exposure of interest.

Eligibility was not restricted by embryo developmental stage at transfer. In the event, all included studies evaluated cleavage-stage transfers, with one study additionally permitting a small proportion of blastocyst transfers [7]. Because contemporary practice increasingly favours blastocyst-stage transfer and elective freeze-all strategies, this feature of the evidence base is flagged here and revisited when appraising indirectness, as it bears directly on the applicability of our findings to current practice.

Information Sources and Search Strategy

We searched PubMed, Scopus, Web of Science, and Embase from database inception to the search date, without language restriction. The search combined controlled vocabulary and free-text terms across three concepts: uterine peristalsis (uterine peristalsis, uterine contractility, uterine contractions, endometrial wave-like activity, junctional zone contractions, subendometrial contractility, endometrial peristaltic waves); assisted reproduction (in vitro fertilisation, IVF, ICSI, intracytoplasmic sperm injection, assisted reproductive technology, embryo transfer, frozen embryo transfer); and outcome (clinical pregnancy, pregnancy rate, implantation, implantation rate, live birth). The three concept blocks were combined with the Boolean operator AND and terms within each block with OR. Reference lists of all included studies and of prior reviews [8,9] were hand-searched, and forward citation tracking was performed. The full electronic search strategy for each database is provided in the Appendix.

Study Selection and Data Extraction

Records were deduplicated and screened by title and abstract, with potentially eligible reports retrieved for full-text assessment against the criteria in Table 1. Two reviewers independently screened records and extracted data, with disagreements resolved by discussion.

For each included study we extracted first author, publication year, country, study design, recruitment period, sample size, participant age, inclusion and exclusion criteria, cycle type, embryo stage, ultrasound modality, recording duration, observer blinding, timing of peristalsis assessment, the frequency threshold used to define high versus low peristalsis, the definition of clinical pregnancy, and the number of clinical pregnancies and total cycles in each stratum. Where studies reported multiple ordered strata, these were collapsed at the study's principal reported threshold to yield a dichotomous contrast. In one study [4], event numbers were back-calculated from published rounded percentages; its influence was examined by sensitivity analysis. All counts were reconciled against published totals.

Risk of Bias Assessment

Because all eligible studies were non-randomised follow-up studies of an exposure rather than of an intervention, risk of bias was assessed using Risk Of Bias In Non-randomised Studies - of Exposures (ROBINS-E) [11], in preference to instruments developed for intervention studies or for generic study-quality scoring. Each study was appraised across seven domains: bias due to confounding; bias arising from measurement of the exposure; bias in selection of participants into the study; bias due to post-exposure interventions; bias due to missing data; bias arising from measurement of the outcome; and bias in selection of the reported result. Domain-level judgements of low risk, some concerns, or high risk were combined into an overall judgement, with the overall rating determined by the least favourable domain. Two reviewers assessed each study independently, resolving disagreements by consensus.

Statistical Analysis

The primary outcome was clinical pregnancy, defined as ultrasonographic visualisation of at least one intrauterine gestational sac. The effect measure was the odds ratio for clinical pregnancy in women with high versus low uterine peristaltic frequency, such that an OR below 1.0 indicates that high peristalsis is associated with reduced odds of pregnancy. Study-level ORs and their standard errors were computed from two-by-two tables on the natural logarithmic scale; a continuity correction of 0.5 would have been applied to all cells of any table containing a zero cell, though no such table arose in the primary analysis.

Effect estimates were pooled using the DerSimonian-Laird random-effects model [12], chosen a priori because the included studies differed in cycle type, ultrasound modality, timing of assessment, and exposure threshold, making a common true effect implausible. Heterogeneity was quantified using Cochran's Q, the between-study variance τ², and the I² statistic [13]. Because I² describes the proportion of observed variance attributable to heterogeneity rather than the dispersion of true effects, a 95% prediction interval was also computed to express the range within which the effect in a future comparable study would be expected to fall [14].

Four prespecified sensitivity analyses were performed: restriction to studies using a harmonised threshold of approximately 2 waves/min; sequential leave-one-out omission of each study; comparison of random-effects with fixed-effect estimates; and reintroduction of the protocol-excluded study in which peristalsis was measured during a preceding natural cycle before a mock transfer, in order to quantify the influence of the stricter exposure definition adopted here. Two prespecified subgroup analyses examined cycle type (fresh, frozen-thawed, mixed) and exposure threshold (two versus three waves/min), with differences between subgroups tested by Cochran's Q statistic for subgroup differences.

Small-study effects were examined by visual inspection of a funnel plot of log odds ratio against standard error and by Egger's regression test of the intercept [15]. Because fewer than 10 studies were available, these assessments are acknowledged to have very low power and are reported for completeness rather than as evidence of the absence of bias. More generally, because only a small number of studies were anticipated, the subgroup analyses, funnel-plot assessment, and sensitivity analyses were prespecified as exploratory and hypothesis-generating rather than confirmatory, and are interpreted with corresponding caution throughout.

Certainty of evidence for the primary outcome was rated using the Grading of Recommendations Assessment, Development and Evaluation (GRADE) framework [16]. As a body of observational evidence, certainty began at low and was assessed for downgrading on risk of bias, inconsistency, indirectness, imprecision, and publication bias, and for upgrading on large magnitude of effect, dose-response gradient, and the direction of plausible residual confounding.

All analyses were performed in Python 3 (Python Software Foundation) using NumPy and SciPy, with the random-effects estimator implemented directly from published formulae and independently cross-validated against a manual inverse-variance computation. Two-sided P values below 0.05 were considered statistically significant. Effect estimates are reported to two decimal places and heterogeneity statistics to one.

Results

Study Selection and Characteristics

**Figure 1:**
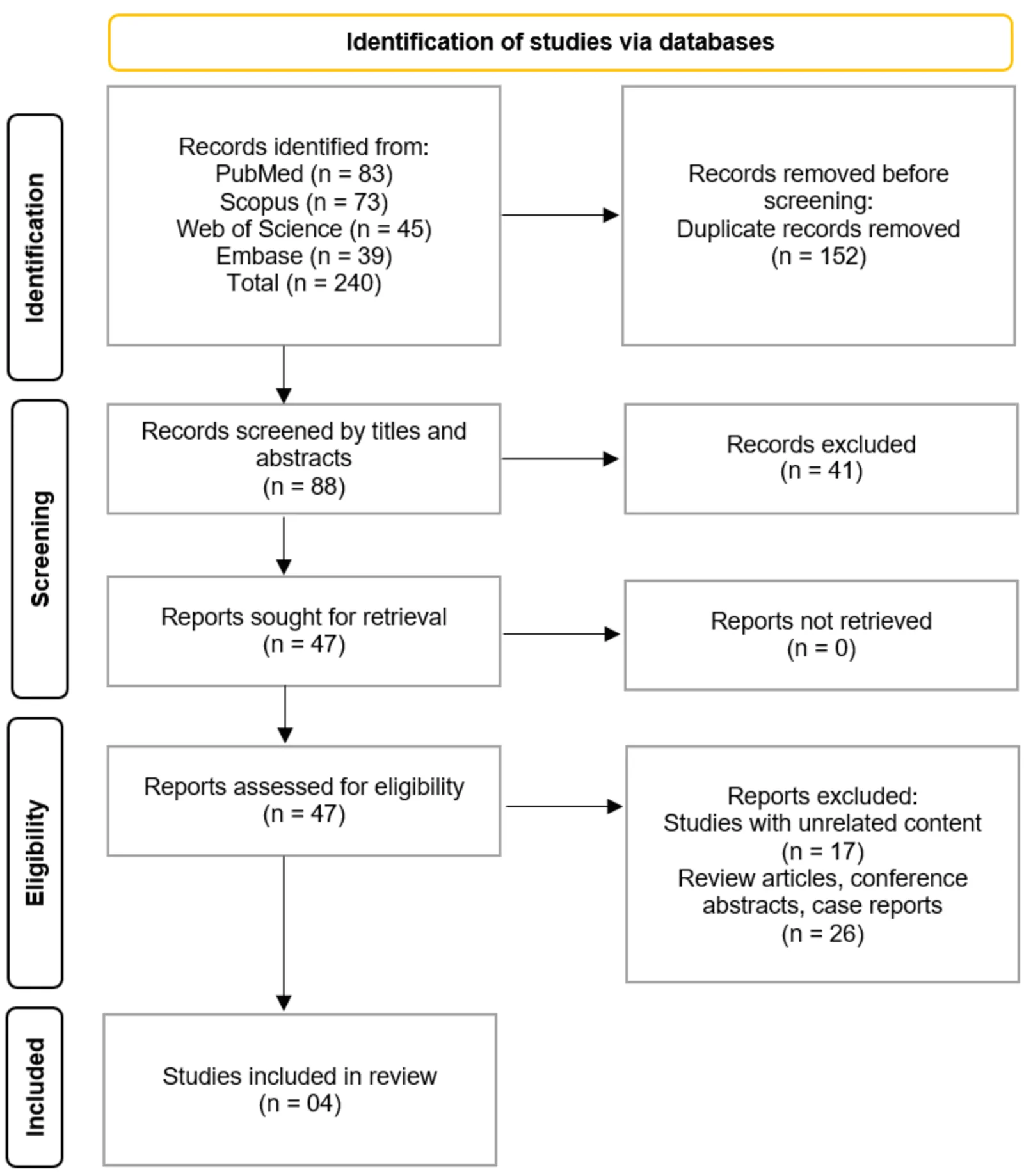
PRISMA 2020 flow diagram of study identification, screening, and inclusion PRISMA: Preferred Reporting Items for Systematic Reviews and Meta-Analyses [10]

The study selection process followed PRISMA 2020 guidelines and is summarised in Figure 1. A systematic search of PubMed, Scopus, Web of Science, and Embase yielded 240 records. After removal of 152 duplicates, 88 records were screened by title and abstract, of which 41 were excluded. All 47 reports sought for retrieval were obtained and assessed for full-text eligibility; of these, 17 were excluded for unrelated content and 26 as reviews, conference abstracts, or case reports. Four studies [4-7] met all eligibility criteria and were included in both the qualitative and quantitative synthesis.

One report warrants specific comment because it has been included in a previous meta-analysis [9] but was excluded here. Zhu et al. studied 112 women who underwent a mock embryo transfer during the natural cycle preceding their IVF treatment cycle, with peristalsis recorded three days after ovulation [17]. Although 89 of these women subsequently underwent transfer and 40 achieved clinical pregnancy, the exposure was measured neither during the transfer cycle nor before an actual transfer, and the authors reported no difference in peristaltic frequency between women who conceived and those who did not (before mock transfer, 2.21 versus 2.12 waves/min, P = 0.903; after mock transfer, 3.22 versus 3.04 waves/min, P = 0.402). The authors further stated that the wide disparity in subgroup sizes and residual confounding rendered the corresponding correlation analysis invalid. This report was therefore excluded from the primary analysis and examined only as a prespecified sensitivity analysis.

Characteristics of the four included studies are presented in Table 2. All were prospective cohort studies, published between 1998 and 2023, and conducted in France [4], China [5], Hong Kong [6], and Iran [7]. Together they contributed 863 embryo transfer cycles, ranging from 68 to 292 cycles per study. All four restricted enrolment to women with a morphologically normal uterus, and all excluded or did not enrol women with recognised uterine pathology; Chung et al. applied the most explicit exclusion criteria, systematically excluding congenital uterine anomaly, myoma, adenomyosis, endometrial polyp, hydrosalpinx, and recurrent implantation failure [6]. Embryo transfer was performed at cleavage stage in all studies. Cycle type differed: two studies enrolled fresh cycles exclusively [4,6], one enrolled artificial frozen-thawed cycles exclusively [7], and one enrolled a mixture of fresh, natural frozen-thawed, and artificial frozen-thawed cycles [5].

**Table 2:** Characteristics of the four included studies

Study	Country	Design	Cycles (n)	Age (years)	Cycle type	Embryo stage	Key eligibility restrictions
Fanchin et al., 1998 [4]	France	Prospective cohort	220	Median 31-33	Fresh	Cleavage (day 2)	Age ≤38; normal uterus on hysteroscopy; ≥3 good-quality embryos
Zhu et al., 2014 [5]	China	Prospective cohort	292	29.1 ± 3.5	Fresh and frozen-thawed	Cleavage (day 3)	Age 20-35; normal uterus; 2 good-quality embryos; atraumatic transfer
Chung et al., 2017 [6]	Hong Kong	Prospective cohort	283	34.6-35.1 ± 3.0–3.4	Fresh	Cleavage (day 3)	Age <40; no uterine pathology or hydrosalpinx; no recurrent implantation failure
Javedani Masroor et al., 2023 [7]	Iran	Prospective cohort	68	34.5 ± 5.6	Artificial frozen-thawed	Cleavage or blastocyst (day 3-5)	Age 25-40; normal uterus; ≥2 good-quality embryos; no recurrent implantation failure

Methods of exposure assessment are detailed in Table 3. Three studies used transvaginal ultrasonography [4,5,7] and one used the transabdominal route, explicitly chosen to minimise cervical stimulation during repeated measurement [6]. Recording duration was five minutes in three studies [4,5,7] and three minutes in one [6]. Two studies used two independent blinded observers and reported excellent agreement, with an intraclass correlation coefficient of 0.988 [5] and inter-observer kappa of 0.75 [4]; one used two blinded radiologists [7]; and one used a single experienced observer with intra-observer kappa of 0.8 [6]. Timing of assessment ranged from immediately before transfer [4] to approximately one hour before transfer [5,7], with one study anchoring its principal analysis five minutes before transfer and additionally measuring at five and sixty minutes afterwards [6]. These timings are not biologically equivalent, a point we return to in the Discussion.

**Table 3:** Uterine peristalsis assessment and clinical pregnancy outcomes

Study	Modality	Duration	Observers	Timing	Threshold (waves/min)	High: n/N (%)	Low: n/N (%)	OR (95% CI)
Fanchin et al., 1998 [4]	Transvaginal	5 min	2, blinded	Immediately pre-transfer	>3.0	43/167 (25.7)	28/53 (52.8)	0.31 (0.16-0.59)
Zhu et al., 2014 [5]	Transvaginal	5 min	2, blinded	~1 h pre-transfer	>2.0	58/135 (43.0)	98/157 (62.4)	0.45 (0.28-0.73)
Chung et al., 2017 [6]	Transabdominal	3 min	1	−5 min pre-transfer	≥2.0	49/136 (36.0)	70/147 (47.6)	0.62 (0.38-1.00)
Javedani Masroor et al., 2023 [7]	Transvaginal	5 min	2, blinded	~1 h pre-transfer	>2.0	10/40 (25.0)	15/28 (53.6)	0.29 (0.10-0.81)
Total	-	-	-	-	-	160/478 (33.5)	211/385 (54.8)	0.45 (0.32-0.62)

The threshold defining high peristalsis differed across studies and was in every case derived post hoc rather than prespecified. Three studies used a threshold of approximately 2 waves/min [5-7], while the earliest study reported its lowest stratum as ≤3.0 contractions/min and consequently supports dichotomisation only at that value [4]. Chung et al. selected 2.0 because it was the observed median in their cohort [6]. Clinical pregnancy was defined by ultrasonographic visualisation of a gestational sac in all four studies.

Risk of Bias

**Figure 2:**
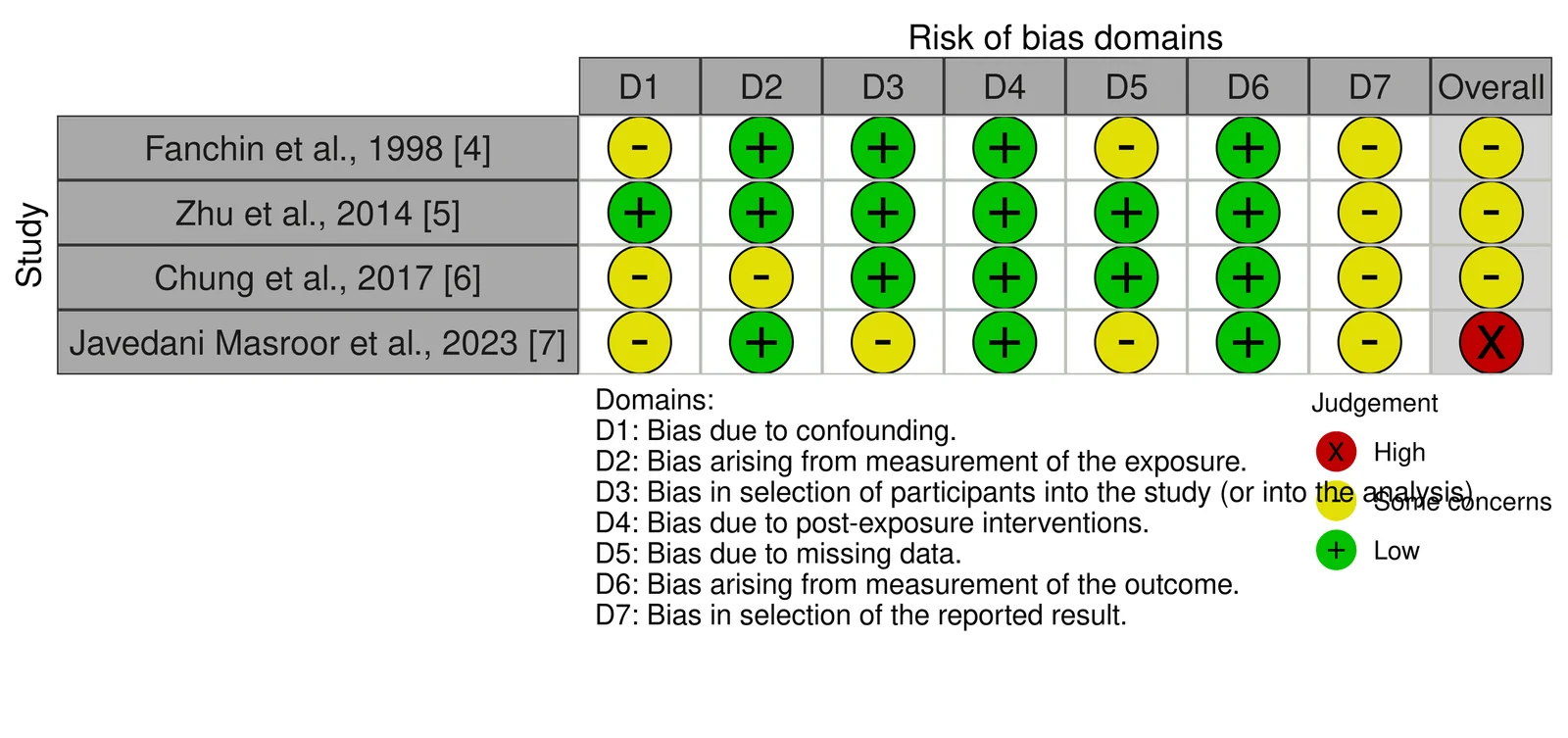
Risk of bias assessed with ROBINS-E Domain-level and overall judgements for each study (D1, confounding; D2, measurement of the exposure; D3, selection of participants; D4, post-exposure interventions; D5, missing data; D6, measurement of the outcome; D7, selection of the reported result). ROBINS-E: Risk Of Bias In Non-randomised Studies - of Exposures [11]

ROBINS-E judgements are summarised in Figure 2. No study was rated at low overall risk of bias. Three studies were judged to raise some concerns [4-6] and one was judged at high risk of bias [7].

Domain-level findings were informative. Measurement of the exposure (D2) was generally well handled: three studies used blinded duplicate observation with quantified agreement and were rated low risk [4,5,7], while Chung et al. raised some concerns owing to single-observer assessment via the transabdominal route, which offers lower resolution of the subendometrial interface [6]. Measurement of the outcome (D6) was rated low risk throughout, since ultrasonographically confirmed clinical pregnancy is an objective endpoint unlikely to be influenced by knowledge of exposure status. Post-exposure interventions (D4) were rated low risk in all studies, as luteal support protocols were standardised within each cohort.

Confounding (D1) was the principal weakness. Only Zhu et al. reported a multivariable model adjusting for age, infertility duration, basal gonadotrophins, oestradiol and progesterone on the day of transfer, endometrial thickness, embryo quality, and cycle type, and was rated low risk [5]. Fanchin et al. demonstrated comparability of stimulation and embryology characteristics across frequency strata but presented no adjusted estimate [4]; Chung et al. adjusted their live birth model but not the clinical pregnancy comparison [6]; and Javedani Masroor et al. fitted a five-covariate model to 68 participants with 25 events, a data-to-parameter ratio that renders the adjusted estimate unstable, as reflected in a confidence interval spanning 1.5 to 79.4 [7]. Selection of the reported result (D7) raised some concerns in every study, since frequency strata were in all cases defined after data collection - described variously as "sorted arbitrarily" [4], "subjectively divided" [5], and derived from the observed median [6] - and no study reported a registered analysis plan specifying the dichotomisation in advance.

Primary Meta-Analysis

Across the four included studies, clinical pregnancy occurred in 160 of 478 cycles (33.5%) in the high-peristalsis group and 211 of 385 cycles (54.8%) in the low-peristalsis group. Every study individually favoured low peristalsis, with study-level ORs ranging from 0.29 to 0.62.

**Figure 3:**
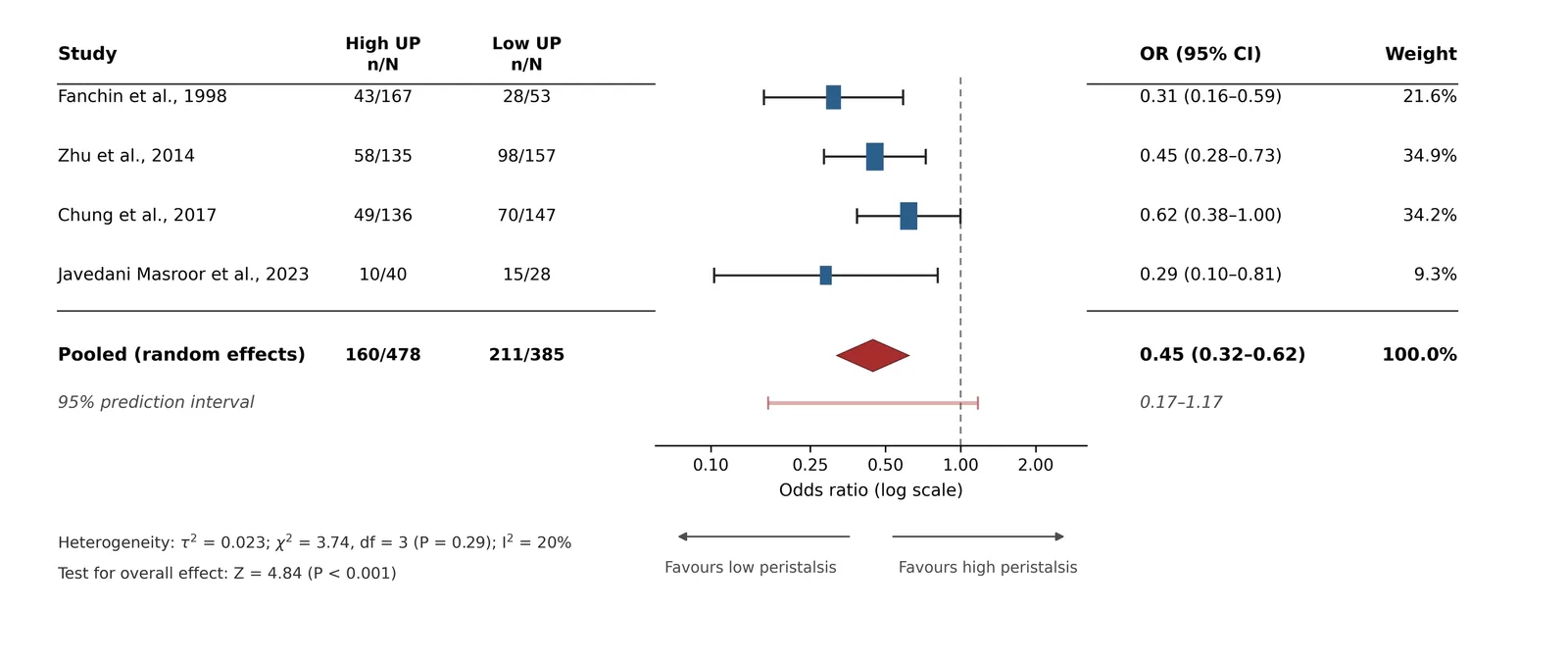
Forest plot of the association between high uterine peristalsis and clinical pregnancy. Squares denote study-level odds ratios with area proportional to random-effects weight; horizontal lines denote 95% confidence intervals; the diamond denotes the pooled random-effects estimate; and the shaded horizontal bar denotes the 95% prediction interval.

Pooling by random-effects meta-analysis yielded an odds ratio of 0.45 (95% CI 0.32-0.62; Z = 4.84, P < 0.001), indicating that high uterine peristaltic frequency around the time of embryo transfer is associated with approximately 55% lower odds of clinical pregnancy (Figure 3). Applied to the observed low-peristalsis baseline risk of 54.8%, this corresponds to an absolute reduction of 197 clinical pregnancies per 1,000 cycles (95% CI 268 fewer to 120 fewer). Because the four studies assessed peristalsis at different moments relative to transfer, this summary estimate necessarily combines exposures that are related but not strictly equivalent, as discussed below.

Heterogeneity was low and not statistically significant (Q = 3.74, df = 3, P = 0.29; I² = 19.8%; τ² = 0.023). The fixed-effect estimate was essentially identical (OR 0.45, 95% CI 0.34-0.60), consistent with limited between-study variance. Notwithstanding this, the 95% prediction interval extended from 0.17 to 1.17 and therefore crossed unity, indicating that despite a precise summary estimate, the effect in a future comparable study could plausibly range from a very large protective association to no association at all. This divergence between a narrow confidence interval and a prediction interval crossing the null is a direct consequence of the small number of studies and is central to interpreting these findings.

Subgroup Analyses

**Figure 4:**
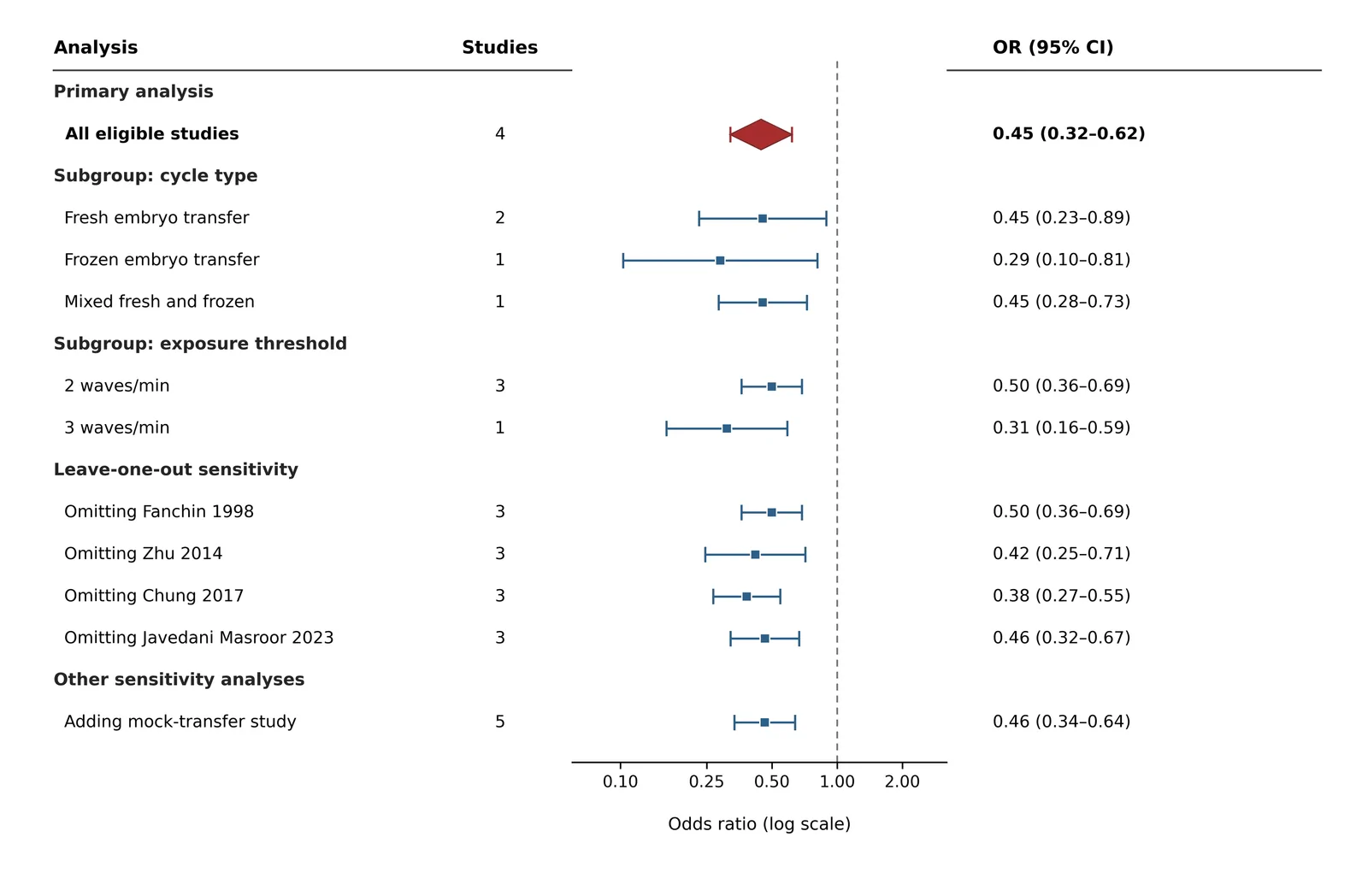
Summary forest plot of prespecified subgroup and sensitivity analyses Fanchin et al. [4]; Zhu et al. [5]; Chung et al. [6]; Javedani Masroor et al. [7]

Results of prespecified subgroup analyses are presented in Table 4 and Figure 4. Stratification by cycle type gave pooled ORs of 0.45 (95% CI 0.23-0.89) for fresh cycles [4,6], 0.29 (95% CI 0.10-0.81) for frozen-thawed cycles [7], and 0.45 (95% CI 0.28-0.73) for the mixed cohort [5], with no evidence of effect modification (Q = 0.64, df = 2, P = 0.72). Stratification by exposure threshold gave 0.50 (95% CI 0.36-0.69) for studies using approximately 2 waves/min [5-7] and 0.31 (95% CI 0.16-0.59) for the single study using 3 waves/min [4]; although the point estimate was more extreme at the higher threshold, as would be expected if a dose-response relationship exists, the difference between subgroups was not statistically significant (Q = 1.70, df = 1, P = 0.19). Both subgroup analyses contain only one or two studies per stratum and, consistent with their prespecified exploratory status, should be regarded as hypothesis-generating rather than confirmatory.

**Table 4:** Primary, subgroup, and sensitivity analyses Test for subgroup differences: cycle type Q = 0.64, df = 2, P = 0.72; threshold Q = 1.70, df = 1, P = 0.19. All subgroup, funnel-plot, and sensitivity analyses were prespecified as exploratory given the small number of studies.

Analysis	Studies (n)	Cycles (n)	OR (95% CI)	P value	I² (%)
Primary (random effects)	4	863	0.45 (0.32-0.62)	<0.001	19.8
Fixed-effect model	4	863	0.45 (0.34-0.60)	<0.001	-
Subgroup: cycle type					
Fresh	2	503	0.45 (0.23-0.89)	0.021	65.5
Frozen–thawed	1	68	0.29 (0.10-0.81)	0.018	-
Mixed	1	292	0.45 (0.28-0.73)	0.001	-
Subgroup: exposure threshold					
~2 waves/min	3	643	0.50 (0.36-0.69)	<0.001	1.4
3 waves/min	1	220	0.31 (0.16-0.59)	<0.001	-
Leave-one-out					
Omitting Fanchin et al. [4]	3	643	0.50 (0.36-0.69)	<0.001	1.4
Omitting Zhu et al. [5]	3	571	0.42 (0.25-0.71)	0.001	46.5
Omitting Chung et al. [6]	3	580	0.38 (0.27-0.55)	<0.001	0.0
Omitting Javedani Masroor et al. [7]	3	795	0.46 (0.32-0.67)	<0.001	32.0
Exposure definition					
Adding mock-transfer study [17]	5	952	0.46 (0.34-0.64)	<0.001	17.8

Sensitivity Analyses

The pooled estimate was stable under all prespecified sensitivity analyses (Table 4, Figure 4). Sequential omission of individual studies produced pooled ORs between 0.38 (95% CI 0.27-0.55, omitting Chung et al.) and 0.50 (95% CI 0.36-0.69, omitting Fanchin et al.), with the confidence interval excluding unity in every iteration. Notably, omission of Fanchin et al. - the only study contributing back-calculated event counts and the only one using a 3 waves/min threshold - reduced heterogeneity to I² = 1.4% while preserving a clearly significant association, indicating that the back-calculation did not drive the result.

Restricting analysis to the three studies employing a harmonised threshold of approximately 2 waves/min [5-7] yielded an OR of 0.50 (95% CI 0.36-0.69; P < 0.001) with near-complete homogeneity (I² = 1.4%; τ² = 0.001; Q = 2.03, df = 2, P = 0.36). This estimate is the most methodologically consistent available and is arguably the most defensible summary of the evidence.

Reintroducing the protocol-excluded mock-transfer study [17] produced a pooled OR of 0.46 (95% CI 0.34-0.64) with I² = 17.8%, demonstrating that the stricter exposure definition adopted in this review did not materially alter the magnitude of the association, although it does alter the interpretation, since that study's own authors found no relationship between peristalsis and pregnancy within their cohort.

Timing of Assessment and Secondary Outcomes

Only one study measured peristalsis at multiple timepoints, precluding meta-analysis of timing effects [6]. Within that study, the association between high contraction frequency and clinical pregnancy strengthened progressively after the transfer procedure: OR 0.62 (95% CI 0.39-1.00) at five minutes before transfer, 0.53 (95% CI 0.33-0.86) at five minutes after, and 0.47 (95% CI 0.29-0.76) at sixty minutes after. The same study was the only one reporting live birth stratified by peristaltic frequency, with an OR of 0.57 (95% CI 0.34-0.94) for high versus low frequency measured before transfer. No pooled estimate for live birth, implantation rate, miscarriage, or ectopic pregnancy could be derived, as fewer than two studies reported each of these outcomes by peristalsis stratum.

Publication Bias and Certainty of Evidence

**Figure 5:**
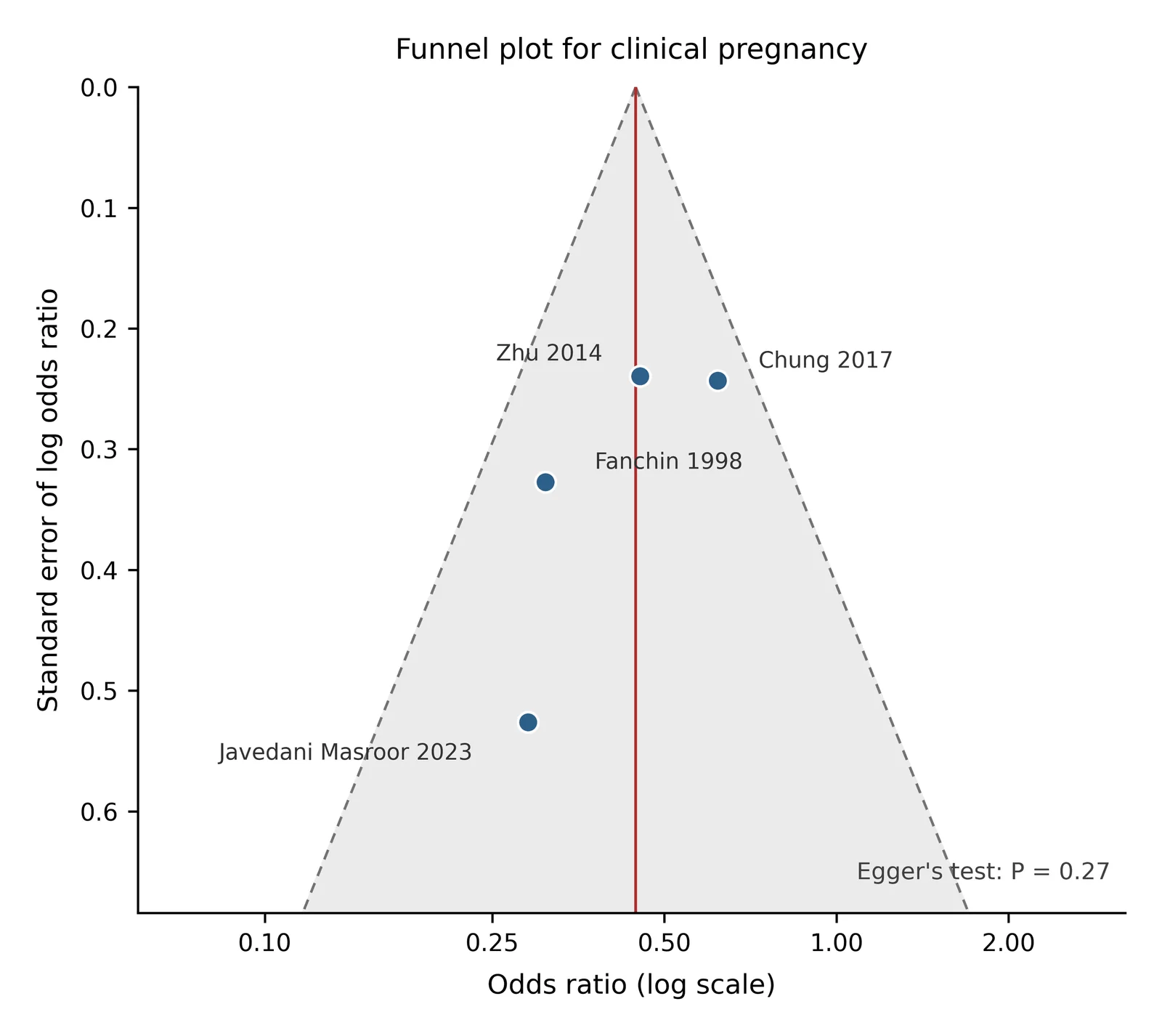
Funnel plot of log odds ratio against standard error, with pseudo-95% confidence region and pooled effect indicated Fanchin et al. [4]; Zhu et al. [5]; Chung et al. [6]; Javedani Masroor et al. [7]

The funnel plot (Figure 5) showed all four studies lying within the pseudo-95% confidence region, and Egger's regression test provided no evidence of asymmetry (intercept −2.62, standard error 1.73; t = −1.52, df = 2, P = 0.27). With only four studies, however, this test has minimal power, and the possibility of small-study effects or selective non-publication of null findings cannot be excluded.

Certainty of evidence for the primary outcome was rated very low (Table 5). Beginning at low certainty as a body of observational evidence, we downgraded once for risk of bias, reflecting that no study was at low overall risk, that confounding adjustment was absent or unstable in three of four studies, and that exposure thresholds were uniformly defined post hoc; and once for indirectness, reflecting exclusive enrolment of cleavage-stage transfers in an era in which blastocyst transfer predominates, the absence of pooled live birth data, and non-comparable exposure definitions across studies. We did not downgrade for inconsistency, as heterogeneity was low and all studies pointed in the same direction, nor for imprecision, as the confidence interval excluded unity and the optimal information size was met. We considered upgrading for large magnitude of effect but elected not to do so, given that unmeasured confounding by ovarian response, endometrial receptivity, and embryo quality could plausibly generate an association of this size in the absence of a causal relationship.

**Table 5:** GRADE summary of findings GRADE: Grading of Recommendations Assessment, Development and Evaluation [16]

Element	Assessment
Outcome	Clinical pregnancy, high versus low uterine peristalsis
Studies / participants	4 prospective cohort studies; 863 embryo transfer cycles
Relative effect	OR 0.45 (95% CI 0.32–0.62)
Absolute effect	548 per 1,000 (low peristalsis) versus 351 per 1,000 (high peristalsis); 197 fewer per 1,000 (268 fewer to 120 fewer)
Starting certainty	Low (observational evidence)
Risk of bias	Serious (−1): no study at low overall risk; confounding unadjusted or unstable in 3 of 4 studies; post hoc thresholds throughout
Inconsistency	Not serious: I² = 19.8%, P = 0.29; all estimates directionally concordant
Indirectness	Serious (−1): cleavage-stage transfers only; no pooled live birth; non-comparable exposure thresholds
Imprecision	Not serious: CI excludes unity; optimal information size met
Publication bias	Undetected but not excludable (4 studies)
Certainty	Very low

Discussion

This systematic review and meta-analysis of four prospective cohort studies comprising 863 embryo transfer cycles found that elevated uterine peristaltic frequency measured during the embryo transfer cycle was associated with approximately 55% lower odds of clinical pregnancy following IVF (pooled OR 0.45, 95% CI 0.32-0.62). Expressed absolutely, this corresponds to roughly 197 fewer clinical pregnancies for every 1,000 cycles. Because this estimate derives entirely from observational cohorts, it should be read as the magnitude of an association and not equated with the effect that an intervention lowering peristalsis would be expected to produce; observational associations of this kind cannot be compared directly with treatment effects from randomised trials. Every included study pointed in the same direction, the association survived every sensitivity analysis we performed, and between-study heterogeneity was low. And yet our overall certainty in this body of evidence is very low. The tension between an apparently robust point estimate and a genuinely fragile evidence base is the central finding of this review, and it deserves to be confronted rather than smoothed over.

The biological rationale for a causal interpretation is coherent and long-standing. Uterine peristalsis in the natural cycle follows a stereotyped trajectory, with frequency peaking around ovulation in a predominantly cervicofundal direction and falling to a nadir during the mid-luteal phase, precisely when a naturally conceived blastocyst would be seeking apposition with the endometrium [1,2]. A mechanically quiescent cavity plausibly permits the sustained embryo-endometrial contact that implantation requires, whereas vigorous contractile activity may displace a transferred embryo from its intended fundal position. Direct experimental support for the displacement hypothesis is substantial. Lesny et al. demonstrated that touching the uterine fundus with a transfer catheter provoked strong contractions capable of expelling instilled fluid into the cervix and fallopian tubes [18], and Zhu et al., using an ultrasound contrast agent as an embryo analogue, showed that peristaltic wave frequency correlated directly with the distance the fluid subsequently migrated, with extrusion from the cavity occurring in 14.5% of transfers and being significantly more common in women with higher peristaltic frequency [17]. The observation that peristalsis physically relocates intrauterine contents is therefore secure; what remains uncertain is whether the degree of relocation observed is sufficient to determine implantation outcome in the human. It must also be kept in view that implantation is a multifactorial process: embryo euploidy, endometrial receptivity, progesterone exposure, embryo transfer technique, and operator experience are all established determinants, and uterine peristalsis is at most one contributor among several. The mechanical emphasis of the present literature reflects the ease of measuring contractions rather than any demonstration that they dominate these other factors, and our findings should be read with that proportion in mind.

Assisted reproduction appears to perturb this physiology in a systematic and clinically relevant way. Zhu et al. demonstrated in a within-woman design that peristaltic frequency was markedly higher in stimulated cycles than in the same women's natural cycles, and - critically - that endometrial activity failed to return to natural-cycle levels before transfer despite supraphysiological progesterone [3]. Fanchin et al. observed an inverse correlation between plasma progesterone at transfer and contraction frequency, consistent with the myorelaxant properties of progesterone, and separately showed that uterine contractility falls substantially by day five after oocyte retrieval, providing a physiological argument for blastocyst rather than cleavage-stage transfer [4,19]. These strands cohere into a plausible mechanistic account: ovarian stimulation elevates oestradiol and thereby uterine contractility; the luteal decline is blunted; cleavage-stage transfer occurs while the uterus remains relatively active; and embryos are transferred into a suboptimal mechanical environment. Our finding that the pooled association was of similar magnitude in fresh and frozen-thawed cycles, however, complicates this account, since frozen cycles ought to be less affected by stimulation-driven hyperperistalsis. The subgroup comparison was severely underpowered, with only one study contributing to the frozen stratum, and should not be over-interpreted in either direction.

Our results are broadly concordant with the two prior syntheses in this field while differing in important methodological respects. Kuijsters et al. concluded that promising early findings had not translated into practice, largely because measurement remained non-standardised [8], and Vidal et al. subsequently pooled five studies to report an OR of 0.52 (95% CI 0.38-0.69) with moderate heterogeneity (I² = 55%) [9]. Our pooled estimate of 0.45 (95% CI 0.32-0.62) is compatible with theirs, and the near-identical conclusion reached from a differently constituted set of studies is reassuring. The heterogeneity, however, differs substantially: we observed I² of 19.8%, falling to 1.4% when analysis was restricted to studies using a harmonised threshold. We attribute this difference to two decisions. First, we excluded from the primary analysis the study in which peristalsis was measured during a natural cycle preceding IVF, before a mock rather than an actual transfer [17]; that study's authors themselves reported no difference in peristaltic frequency between women who conceived and those who did not, and explicitly characterised the corresponding subgroup analysis as invalid. Second, we did not impose a uniform 2 contractions/min dichotomy on a study whose published strata do not permit it, since Fanchin et al. reported their lowest stratum as ≤3.0 contractions/min [4]; we therefore analysed that study at its own threshold. Reassuringly, reintroducing the mock-transfer study altered our pooled estimate only trivially (OR 0.46, 95% CI 0.34-0.64), indicating that these choices refine interpretation rather than reverse conclusions.

Two aspects of our findings temper enthusiasm. The first is the prediction interval, which extended from 0.17 to 1.17 and therefore crossed the null even though the confidence interval around the pooled estimate was narrow and excluded unity. The prediction interval is wider than the confidence interval because it additionally incorporates the estimated between-study heterogeneity (τ²) rather than describing only the precision of the pooled mean; with just four studies, τ² is itself estimated imprecisely, widening the interval further. In practical terms, a future well-conducted cohort finding no association would not be inconsistent with the present evidence, and the prediction interval is the appropriate guard against the well-recognised tendency of small observational meta-analyses to produce confident-looking summaries that larger studies fail to replicate. The second is that every threshold used to define "high" peristalsis in this literature was chosen after the data were collected. Fanchin et al. described their strata as sorted arbitrarily [4]; Zhu et al. described their groups as subjectively divided and derived a cut-off of 2.45 waves/min by receiver-operating-characteristic analysis within the same dataset used to test the association [5]; Chung et al. selected 2.0 because it was the observed median of their cohort [6]; and Javedani Masroor et al. used three strata without stated prior justification [7]. Data-driven dichotomisation is a recognised source of optimism bias, and the consistency of direction across studies does not protect against it, since each study selected its own most discriminating threshold. Until a threshold is prespecified in one cohort and validated in an independent one, a cut-off such as "two contractions per minute" should be regarded as provisional.

The question of when to measure is at least as important as where to draw the threshold, and here the evidence is genuinely thin. Only Chung et al. assessed peristalsis at multiple timepoints, and within that study the association strengthened progressively after the procedure, from an OR of 0.62 immediately before transfer to 0.53 at five minutes and 0.47 at 60 minutes afterwards [6]. The authors observed that the transient post-transfer rise in contraction frequency was significant only among women who did not conceive, and that the single variable independently predicting contraction frequency was the sensation of urinary urgency, implicating bladder overdistension rather than intrinsic uterine pathology [6]. Related work on transfer technique using the embryo-associated air bubble as a marker has similarly emphasised the mechanical determinants of embryo position [20]. More fundamentally, the four pooled studies did not measure peristalsis at a common moment: assessment ranged from immediately before transfer to approximately one hour beforehand, with one study anchoring its principal analysis five minutes before the procedure. Pooling these measurements assumes that peristaltic frequency at these different moments indexes the same underlying exposure, an assumption unlikely to hold exactly given that contractility responds within minutes to catheter manipulation and bladder filling. The summary estimate should therefore be understood as an average over related but non-identical exposures; the harmonised-threshold sensitivity analysis, which happens also to group the more comparably timed studies, offers a partial safeguard, but the absence of post-transfer measurement in three of four studies means the pooled estimate reflects pre-transfer contractility, which may not be the exposure that matters most.

Frequency is, moreover, only one dimension of contractile behaviour. Blank et al., in a pilot study using quantitative ultrasound imaging, suggested that a combination of higher frequency with lower amplitude might actually favour implantation, implying that frequency in isolation may be an incomplete summary of uterine activity [21]. Direction may matter independently: Chung et al. found that live birth rates were highest among women with absent or indeterminate wave direction 60 minutes after transfer [6], while Kim et al., studying intrauterine insemination, reported higher pregnancy rates in association with cervicofundal endometrial movement [22]. Broader evidence that contractile dysfunction is implicated in unexplained infertility [23] suggests that peristalsis is best conceived as a multidimensional phenotype rather than a single scalar, and the near-exclusive focus of the present literature on frequency may have obscured more informative parameters.

The therapeutic corollary - that pharmacologically quietening the uterus should improve outcomes - provides the sternest test of causality, and it has not been passed. The Cochrane review of oxytocin antagonists for assisted reproduction concluded that the evidence was insufficient to establish benefit for live birth or clinical pregnancy, with the available trials small and at risk of bias [24], and a subsequent randomised, double-blind trial of atosiban in women with recurrent implantation failure likewise failed to demonstrate a convincing improvement in outcome [25]. This disconnect admits several readings. Uterine peristalsis may be a marker of an unfavourable uterine or endometrial milieu rather than a cause of implantation failure, in which case suppressing contractions would not be expected to help; alternatively, existing agents may be mistimed, underdosed, or applied to unselected populations rather than to the subset with genuinely elevated contractility. Distinguishing these possibilities requires a trial that uses peristaltic frequency as an enrolment criterion rather than as an afterthought, which to our knowledge has not been performed. In practical terms, therefore, a clinician cannot at present alter management on the basis of uterine peristalsis alone: no validated threshold exists to act upon, no intervention has been shown to improve outcomes in women selected by peristalsis, and measurement is not yet standardised. Peristalsis assessment remains a research tool rather than a clinical decision variable, and the association we have quantified should be regarded as prognostic rather than as an established therapeutic target.

Finally, the applicability of this evidence to contemporary practice is limited in a way that no statistical procedure can repair. All four included studies transferred cleavage-stage embryos, whereas blastocyst transfer and elective freeze-all strategies now predominate in many well-resourced units. Both developments change the uterine environment at the moment of transfer: freeze-all removes the supraphysiological steroid milieu of the stimulated cycle, and blastocyst transfer occurs after contractility has already declined by day five [19]. The exposure contrast that generated these results may therefore be attenuated or even absent in contemporary blastocyst and freeze-all cycles, so the present estimate may not transport to current practice and could overstate what would be observed today. Similarly, ultrasound technology has advanced substantially since 1998, and none of the included studies used three-dimensional imaging, speckle-tracking quantification, or electrohysterography. The evidence base is, in a real sense, describing a version of IVF that is receding.

Limitations

Several limitations qualify our conclusions. First, only four studies met eligibility criteria, and although this reflects deliberate methodological strictness rather than an incomplete search, it limits the reliability of heterogeneity estimation and means that the subgroup analyses, funnel plot, and sensitivity analyses - all prespecified as exploratory - can only be hypothesis-generating; it also leaves the prediction interval crossing unity. Second, no study was at low overall risk of bias, and only one reported an adequately adjusted multivariable estimate [5]; our pooled estimate is therefore based predominantly on unadjusted associations, and residual confounding by embryo quality, ovarian response, endometrial receptivity, and operator skill cannot be excluded. Third, exposure thresholds differed across studies and were uniformly derived post hoc, so the pooled odds ratio represents an average across non-identical exposure contrasts rather than a single well-defined comparison. Fourth, peristalsis was assessed at different times relative to transfer across the included studies, so the pooled exposure is not strictly uniform and the summary estimate combines measurements that are not biologically equivalent. Fifth, event counts for one study were back-calculated from published rounded percentages [4]; although this introduces at most one event of rounding error per stratum and leave-one-out analysis confirmed it did not drive the result, it is a departure from ideal data extraction and is disclosed accordingly. Sixth, we could not pool live birth, implantation rate, miscarriage, or ectopic pregnancy, since fewer than two studies reported each by peristalsis stratum; clinical pregnancy is a surrogate for the outcome patients actually value. Seventh, tests for funnel plot asymmetry are uninformative with four studies, and selective non-publication of null results remains plausible in a field where positive findings have dominated since 1998. Eighth, all included studies enrolled women with a morphologically normal uterus, so these findings cannot be extrapolated to women with adenomyosis, fibroids, or recurrent implantation failure, in whom contractile dysfunction may be both more prevalent and more consequential. Finally, this review was not prospectively registered, which is itself a limitation; and while the review methodology is fully reproducible, the reproducibility of the underlying evidence is separately constrained by heterogeneity in ultrasound modality, recording duration, timing of assessment, and peristalsis thresholds across the primary studies - constraints that attach to the evidence base rather than to the review process.

## Conclusions

High uterine peristaltic frequency measured during the embryo transfer cycle is associated with approximately half the odds of clinical pregnancy in IVF, an association that is directionally consistent across four independent cohorts spanning 25 years, four countries, and both fresh and frozen-thawed protocols, and that is robust to leave-one-out omission and to restriction to a harmonised measurement threshold. This consistency is striking and warrants continued investigation. It does not, however, warrant clinical adoption. The certainty of the underlying evidence is very low: no study was at low risk of bias, exposure thresholds were defined post hoc in every case and assessed at differing times relative to transfer, live birth could not be pooled, all transfers were at cleavage stage, and the prediction interval crossing unity means a future study finding no association would be entirely compatible with current knowledge. Interventional trials of contractility-suppressing agents in unselected populations have not demonstrated benefit, leaving open the possibility that peristalsis is a marker of an unfavourable uterine environment rather than a cause of implantation failure. In current practice, therefore, uterine peristalsis alone cannot guide clinical decisions. The priorities for the field are correspondingly clear: consensus on a standardised measurement protocol specifying route, duration, timing, and observer requirements; prospective validation of a prespecified frequency threshold in an independent cohort; extension of measurement to blastocyst-transfer and freeze-all cycles and to post-transfer timepoints; incorporation of amplitude and direction alongside frequency; and, ultimately, a randomised trial in which elevated peristalsis is the enrolment criterion and live birth is the endpoint. Until these are delivered, uterine peristalsis should be regarded as a biologically plausible and clinically intriguing prognostic marker whose value remains unproven.

## Appendices

**Table 6:** Search Strategy for Databases

Database	Search strategy
PubMed/MEDLINE	#1 Uterine peristalsis/contractility: "Uterine Contractions"[Mesh] OR "Myometrium"[Mesh] OR "Muscle, Smooth"[Mesh] OR ((uterine[tiab]) AND (contraction*[tiab] OR contractility[tiab] OR peristalsis[tiab] OR peristaltic[tiab] OR "uterine wave*"[tiab] OR "uterine activ*"[tiab] OR "uterine movement*"[tiab] OR "uterine motility"[tiab] OR "uterine activity"[tiab] OR "endometrial wave*"[tiab] OR "endometrial movement*"[tiab] OR "junctional zone contraction*"[tiab] OR "junctional zone peristalsis"[tiab] OR "junctional contraction*"[tiab] OR "subendometrial contraction*"[tiab] OR "sub-endometrial contraction*"[tiab] OR "subendometrial peristalsis"[tiab] OR "sub-endometrial peristalsis"[tiab] OR "myometrial contraction*"[tiab] OR "myometrial peristalsis"[tiab])) AND #2 IVF/embryo transfer: "Fertilization in Vitro"[Mesh] OR "Embryo Transfer"[Mesh] OR "Reproductive Techniques, Assisted"[Mesh] OR ("in vitro fertilization"[tiab] OR "in-vitro fertilization"[tiab] OR IVF[tiab] OR ICSI[tiab] OR "intracytoplasmic sperm injection"[tiab] OR "assisted reproduction"[tiab] OR "assisted reproductive technolog*"[tiab] OR "fertility treatment*"[tiab] OR "infertility treatment*"[tiab] OR "embryo transfer"[tiab] OR "embryo implantation"[tiab] OR "embryo replacement"[tiab] OR "blastocyst transfer"[tiab] OR "cleavage-stage embryo transfer"[tiab] OR "frozen embryo transfer"[tiab] OR FET[tiab] OR "fresh embryo transfer"[tiab])
Scopus	TITLE-ABS-KEY((uter* W/3 (contraction* OR contractility OR peristalsis OR peristaltic OR motility OR activit* OR wave* OR movement*)) OR "uterine peristalsis" OR "uterine contractions" OR "uterine contractility" OR "uterine activity" OR "uterine motility" OR "endometrial wave*" OR "endometrial movement*" OR "junctional zone contraction*" OR "junctional zone peristalsis" OR "junctional contraction*" OR "subendometrial contraction*" OR "sub-endometrial contraction*" OR "subendometrial peristalsis" OR "sub-endometrial peristalsis" OR "myometrial contraction*" OR "myometrial contractility" OR "myometrial peristalsis") AND TITLE-ABS-KEY("in vitro fertilization" OR "in-vitro fertilization" OR IVF OR ICSI OR "intracytoplasmic sperm injection" OR "assisted reproduction" OR "assisted reproductive technolog*" OR "assisted reproductive treatment*" OR "fertility treatment*" OR "infertility treatment*") AND TITLE-ABS-KEY("embryo transfer" OR "embryo implantation" OR "embryo replacement" OR "blastocyst transfer" OR "cleavage-stage embryo transfer" OR "cleavage stage embryo transfer" OR "frozen embryo transfer" OR FET OR "fresh embryo transfer")
Web of Science Core Collection	TS=((uter* NEAR/3 (contraction* OR contractility OR peristalsis OR peristaltic OR motility OR activit* OR wave* OR movement*)) OR "uterine peristalsis" OR "uterine contractions" OR "uterine contractility" OR "uterine activity" OR "uterine motility" OR "endometrial wave*" OR "endometrial movement*" OR "junctional zone contraction*" OR "junctional zone peristalsis" OR "junctional contraction*" OR "subendometrial contraction*" OR "sub-endometrial contraction*" OR "subendometrial peristalsis" OR "sub-endometrial peristalsis" OR "myometrial contraction*" OR "myometrial contractility" OR "myometrial peristalsis") AND TS=("in vitro fertilization" OR "in-vitro fertilization" OR IVF OR ICSI OR "intracytoplasmic sperm injection" OR "assisted reproduction" OR "assisted reproductive technolog*" OR "assisted reproductive treatment*" OR "fertility treatment*" OR "infertility treatment*") AND TS=("embryo transfer" OR "embryo implantation" OR "embryo replacement" OR "blastocyst transfer" OR "cleavage-stage embryo transfer" OR "cleavage stage embryo transfer" OR "frozen embryo transfer" OR FET OR "fresh embryo transfer")
Embase (Ovid)	#1 Uterine peristalsis: exp uterus contraction/ OR exp uterine muscle/ OR exp myometrium/ OR ((uterine adj3 (contraction* OR contractility OR peristalsis OR peristaltic OR motility OR activit* OR wave* OR movement*)).ti,ab,kw.) OR uterine peristalsis.ti,ab,kw. OR uterine contractions.ti,ab,kw. OR uterine contractility.ti,ab,kw. OR uterine activity.ti,ab,kw. OR uterine motility.ti,ab,kw. OR endometrial wave*.ti,ab,kw. OR endometrial movement*.ti,ab,kw. OR junctional zone contraction*.ti,ab,kw. OR junctional zone peristalsis.ti,ab,kw. OR junctional contraction*.ti,ab,kw. OR subendometrial contraction*.ti,ab,kw. OR sub-endometrial contraction*.ti,ab,kw. OR subendometrial peristalsis.ti,ab,kw. OR sub-endometrial peristalsis.ti,ab,kw. OR myometrial contraction*.ti,ab,kw. OR myometrial contractility.ti,ab,kw. OR myometrial peristalsis.ti,ab,kw. AND #2 IVF/embryo transfer: exp in vitro fertilization/ OR exp assisted reproductive technology/ OR exp embryo transfer/ OR ("in vitro fertilization" OR "in-vitro fertilization" OR IVF OR ICSI OR "intracytoplasmic sperm injection" OR "assisted reproduction" OR "assisted reproductive technolog*" OR "assisted reproductive treatment*" OR "fertility treatment*" OR "infertility treatment*").ti,ab,kw. OR embryo transfer.ti,ab,kw. OR embryo implantation.ti,ab,kw. OR embryo replacement.ti,ab,kw. OR blastocyst transfer.ti,ab,kw. OR cleavage-stage embryo transfer.ti,ab,kw. OR frozen embryo transfer.ti,ab,kw. OR fresh embryo transfer.ti,ab,kw. OR FET.ti,ab,kw.
